# Network and parasitological analyses reveal latitudinal gradient in bats‐ectoparasitic fly interactions across the Neotropic

**DOI:** 10.1002/ece3.10527

**Published:** 2023-09-15

**Authors:** Luana S. Biz, Vinicius A. G. Bastazini, Fernando Carvalho, Maria João Ramos Pereira

**Affiliations:** ^1^ Programa de Pós‐graduação em Ecologia Instituto de Biociências, Universidade Federal do Rio Grande do Sul (UFRGS) Porto Alegre Brazil; ^2^ Departamento de Zoologia, Instituto de Biociências, Bird and Mammal Evolution, Systematics and Ecology Laboratory Universidade Federal do Rio Grande do Sul Porto Alegre Brazil; ^3^ ‘Rui Nabeiro’ Biodiversity Chair University of Évora. Rua Dr. Joaquim Henrique da Fonseca Évora Portugal; ^4^ MED – Mediterranean Institute for Agriculture, Environment and Development & CHANGE – Global Change and Sustainability Institute, Institute for Advanced Studies and Research University of Évora Évora Portugal; ^5^ Programa de Pós‐Graduação em Ciências Ambientais da Universidade do Extremo Sul Catarinense (UNESC) Criciúma Brazil; ^6^ Laboratório de Zoologia e Ecologia de Vertebrados (LABZEV) da Universidade do Extremo Sul Catarinense (UNESC) Criciúma Brazil; ^7^ Centro de Estudos do Ambiente e do Mar (CESAM) Universidade de Aveiro Aveiro Portugal

**Keywords:** biogeography, Chiroptera, macroecology, Nycteribiidae, parasitology, Streblidae

## Abstract

Ecological interactions between parasites and their hosts play a fundamental role in evolutionary processes. Selection pressures are exerted on parasites and their hosts, usually resulting in high levels of specificity. Such is the case of ectoparasitic bat‐flies, but how large‐scale spatial gradients affect the dynamics of their interactions with their bat hosts is still unknown. In the present study, we investigated interaction patterns between bats and their ectoparasitic flies (Streblidae and Nycteribiidae), both presenting their peak of diversity in the Neotropical region, along a latitudinal gradient. Using network analyses and parasitic indices, grounded on the latitudinal diversity gradient pattern, we evaluated how spatial gradients affect species interactions and parasitic indices at the biogeopraphic scale, with increasing species richness in interaction networks closer to the tropics, leading to increases in network modularity, size, and specialization, and to a decrease in nesting and connectivity. We conducted a literature review, focusing on studies done in the Neotropical region, and own data. We obtained a bat richness of 97 species parasitized by 128 species of ectoparasitic flies, distributed into 57 interaction networks between latitudes 29° S and 19° N in the Neotropic. Network metrics and parasitic indices varied along the latitudinal gradient, with changes in the richness of bats and their ectoparasitic flies and in the structure of their interactions; network specialization, modularity, and connectance increase with latitude, while network size decreases with latitude. Regions closer to the equator had higher parasite loads. Our results show that interaction network metrics present a latitudinal gradient and that such interactions, when observed at a local scale, hide variations that only become perceptible at larger scales. In this way, ectoparasites such as bat flies are not only influenced by the ecology and biology of their hosts, but also by other environmental factors acting directly on their distribution and survival.

## Graphical abstract

The article brings unpublished information about the interactions and parasitic indices of bats and their flies. Showing that the specialization of interactions increases with the distance from the tropics.

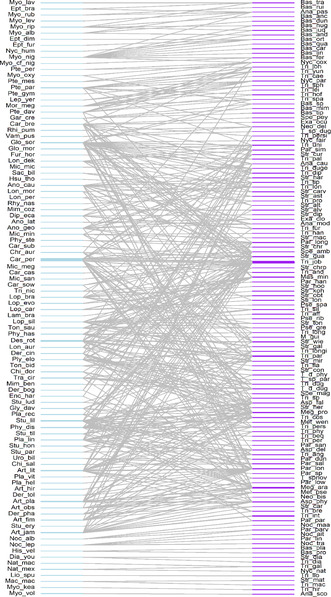

## 1 INTRODUCTION

Ecosystems are composed of a wide diversity of ecological interactions at different trophic levels (Ricklefs & Relyea, 2016). Such interactions act on the dynamics and stability of the environments (Xing & Fayle, 2021), promoting biodiversity. The understanding of the time, space, and mode of these interactions is still incipient, despite being fundamental for the understanding of ecological patterns and processes and for the design of truly holistic conservation measures. In this context, ecological network analyses constitute a fundamental tool for a comprehensive and integrated understanding of ecological systems (Delmas et al., 2019; Guimarães, 2020; Júnior et al., 2020), particularly the structural and functional dynamics of ecological interactions (Olesen et al., 2007; Vázquez et al., 2009). The interaction network approach has been known in the field of ecology since at least the 1940s, with the beginning of classic research on antagonistic relationships (Melo, 2010). However, only recently, has this tool become popular among ecologists (Dáttilo & Rico‐Gray, 2018; Júnior et al., 2020; Pellissier et al., 2018).

The relationship between parasites and their hosts deserves full attention in ecology (Gómez & Nichols, 2013). Indeed, biodiversity is driven and sustained by eco‐evolutionary processes (Solé, 2022), in which parasites play a fundamental role. In 1973, the evolutionary biologist Leigh Van Valen formulated the Red Queen hypothesis, where he postulated that interacting species in ecological networks are in constant coevolution (Solé, 2022; Van Valen, 1973). Parasites are the commonest life form found on Earth (Poulin & Morand, 2000) and are in a perpetual arms race with their hosts (Papkou et al., 2016), making them key players in maintaining biological diversity. Parasites play fundamental roles at the individual level, acting on the host's immune response, at the population level, for example, structuring host population density and affecting survival rates, and at the ecosystem level, acting for instance on nutrient cycles (Ebert et al., 2000; Gómez et al., 2012; Speer et al., 2020).

Among the parasitic systems found in mammals, bats (Chiroptera) and their ectoparasitic flies (Streblidae and Nycteribiidae), known as “bat‐flies,” are a model system. This is due, on the one hand, to the diversity of biological and ecological characteristics of the Chiroptera, reflected in its taxonomic and ecomorphological variety, including body size, dietary and foraging strategies, roosting ecology, social systems, and colony size, as well as to the wide geographic distribution of the group (Patterson et al., 2007). On the other hand, the system is a model also because of the evolutionary characteristics of ectoparasites, particularly their high specificity — about 70% to 90% of bat flies in the Neotropic parasitize a specific species or genus of bat (Dick, 2007; Dick & Gettinger, 2005; Marshall, 1982) — adenotrophic viviparity, and the presence of winged, brachypterous, and wingless species (Marshall, 1982). Those diverse attributes found in the two groups contribute to different infestation patterns (Hiller et al., 2021).

Understanding the patterns of these associations at the biogeographic scale is a fundamental ecological question, as different features along environmental gradients shape the richness and structure of interactions between species. Especially for bats and their ectoparasitic flies, attributes such as roosting ecology and colony size can be factors structuring these interactions, as roosts are crucial for the reproductive success and survival of these bat flies (Urbieta et al., 2022). Unavoidably, bat occupancy is dependent on adequate roost availability in the surrounding environment (Guimarães & Ferreira, 2014), so at the biogeographic scale modulations in these interactions along the latitudinal gradient may be revealed, which at smaller scales are potentially imperceptible.

A trend toward increasing ectoparasite community richness (Bush et al., 1997) and parasite loads for bat flies toward the equator is to be expected, due to the availability of many, and particularly large, caves in the tropical region (Barros et al., 2021; Ladle et al., 2012; Tanalgo et al., 2022; Vargas‐Mena et al., 2020). These underground roosts, when used by bats are known as “bat caves” (Ito et al., 2022). Among these, there are “hot caves”, which are certain underground chambers presenting consistently high temperatures generated by the presence of very large bat colonies, together with specific speleological characteristics (Ito et al., 2022; Ladle et al., 2012). Several bats roosting in such caves have geographic ranges restricted to the tropical region and are found exclusively or predominantly in those “hot caves”, as is the case of the species of the *Pteronotus* genus in the Neotropic (Ito et al., 2022; Ladle et al., 2012; Tanalgo et al., 2022). The combination of those factors should promote greater diversity of bat flies, fitting the general latitudinal diversity gradient pattern. The latitudinal gradient in species richness, with an increasing number of species toward the equator, is one of the most accepted patterns in ecology (Mittelbach et al., 2007; Rohde, 1992), and is more clearly observed at larger scales than on smaller scales (Mittelbach et al., 2007). In contrast, recent large‐scale studies on latitudinal gradients in biotic interactions have shown mixed results, not supporting the idea that specialization between species is always higher in the tropics (Moles & Ollerton, 2016). Studying such wide‐scale associations and comparing interaction networks in different environments along the latitudinal gradient may help us recognize the environmental factors of which the latitude is only a proxy structuring those interactions (Júnior et al., 2020; Pellissier et al., 2018; Xing & Fayle, 2021), and how those networks are shaped concomitantly with changes in the richness of species of both parasites and hosts.

The Neotropical region, extending from Central Mexico and the Caribbean Islands to southern South America (Dinerstein et al., 2017), is characterized by its incredible biodiversity (Dinerstein et al., 2017). Here bats present their peak of taxonomic and eco‐morphological diversity (Aguirre, 2002; Sampaio et al., 2003). Indeed, at the local level, we may find up to 117 bat species, with most environments harboring over 50 species per 25 km^2^ (Delgado‐Jaramillo et al., 2020; Fischer et al., 2018). Furthermore, particularly in neotropical rainforests, bats account for about 40% of the mammal species present (Aguirre, 2002; Emmons & Feer, 1997; Sampaio et al., 2003). With no surprise, this is reflected in the richness and diversity of their ectoparasitic flies, which is positively correlated with that of their hosts (Dick & Gettinger, 2005).

Here, we hypothesize that the patterns of interactions between bats and their ectoparasitic flies are shaped by the latitudinal diversity gradient. Specifically, we expect a modification of the structure of the interaction networks between bats and their ectoparasitic flies throughout the Neotropic. As we approach the tropics, interaction networks should become richer in species, following the typical latitudinal diversity gradient; as a result, this should lead to an increase in network modularity, size, and specialization, and a decrease in nestedness and connectance. This is due to the fact that parasite–host interaction networks present a pattern where the richer the network, that is, the greater its size, the greater its modularity and specialization, and consequently, the lesser its nestedness and connectance. We expect this pattern for flies because they are highly specialized on their host species (Dick, 2007; Dick & Gettinger, 2005; Marshall, 1982). Second, we expect greater richness and ectoparasitic load at lower latitudes. This should reflect the greater diversity of bats and the presence of larger colonies at lower latitudes in the Neotropical region (De Oliveira et al., 2018), supported by an apparent high availability of hot caves (Ladle et al., 2012) as reflected by recent findings in northeastern Brazil (e.g., Vargas‐Mena et al., 2018).

## 2 MATERIALS AND METHODS

### 2.1 Data collection

The present study was based on scientific publications on bats and the ectoparasitic bat‐flies of the Streblidae and Nycteribiidae (Data S1). We conducted a bibliometric search for articles indexed in the databases: Scopus, Web of Science, ScienceDirect, and SciELO, using the keywords “bats” or “Chiroptera”, and “ectoparasite” or “Nycteribiidae” or “Streblidae”. We added the retrieved manuscripts to Mendeley, in which we excluded duplicates (articles indexed in more than one database). After compiling the articles, we selected those including studies done in the Neotropical region, containing information on species richness and abundance for ectoparasites and bats, and latitude data. For those manuscripts where the geographic location was not given in detail, we extracted approximated geographic coordinates using Google Earth. For manuscripts with more than one sampling site, we separated them into different datasets. The data used in the research comprised articles published from 1999 to 2021. We also included in the database own data collected between 2016 and 2022 at four sites in the southern Brazilian Atlantic Forest; each site was sampled monthly for 1 year using 10 × 10 m mist nets. All captured bats were identified at the species level, following the taxonomic keys of Miranda et al. (2011) and Diaz et al. (2016), as well as their biometric data and collected ectoparasites. For the collection of ectoparasites, each bat was visually inspected, the ectoparasites, when present, were collected with the aid of fine‐tipped tweezers, and preserved in 70% alcohol. All ectoparasites were identified at species level following the taxonomic keys of Guerreiro (1993, 1994, 1995a, 1995b, 1996) and Graciolli (2004).

### 2.2 Structuring of interaction networks

For each dataset, we built weighted matrices, based on the frequency of occurrence of the interactions between bat flies and their bat hosts. For each local network, we plotted the interaction networks, calculating the following indices: (i) specialization (H2): depicting the level of exclusivity of the ecological interactions, measuring niche complementarity between species, with values varying from 0 to 1, from the least to the most specialized the network (Blüthgen, 2010); (ii) modularity: evaluating the way in which nodes are partitioned into separate subsets within the network as a whole, using the “Beckett” metric (Beckett, 2016; Mello et al., 2016); (iii) nestedness (NODF metric): evaluating how closely a matrix comes to being perfectly nested, that is the overlap of more specialist species with more generalist species, that is, the tendency of more specialized species to interact with a subset of the interaction partners of more generalized species, varying from 0 to 100, respectively from non‐nested to fully nested networks (Almeida‐Neto et al., 2008); (iv) connectance: evaluating the proportion between the interactions found in the network and all the possible interactions (Mello et al., 2016); and (v) network size: the number of vertices (i.e., species) found in the network (Mello et al., 2016). To calculate the indices and to generate the networks, we used the *vegan*, *bipartite*, and *network* packages (Butts, 2020; Dormann et al., 2009; Oksanen et al., 2018) in the R environment (R Core Team, 2018).

### 2.3 Parasitic indices

In parasitology, for each parasite sample, usually, the sampling units are the individual hosts (Reiczigel et al., 2019). Thus, the presence of parasites at the individual or population level creates patterns that may be described by specific parasitic indices describing aspects of the parasitic infections (Reiczigel et al., 2019). Here, for each set of data, that is for each sampling location, we used two of the mainly used parasitological indices: (i) general prevalence of infestation (P): the proportion of infected hosts in relation to the total set of hosts sampled and (ii) mean infestation intensity (MI): the arithmetic mean of parasites found in a set of infected hosts, excluding parasite‐free hosts. The two indices were calculated using the Quantitative Parasitology 1.0.15 environment (QPWeb, Reiczigel et al., 2019).

### 2.4 Evaluating the influence of latitude on network structure, richness, and parasitic indices

To analyze whether there was a difference in the structure of the interaction networks along the latitudinal gradient, we used generalized linear models (GLMs). We used the structural network metrics (specialization, modularity, nestedness, connectance, and network size) as response variables, and latitude as the predictor variable. We also used network size as a predictor of specialization, modularity, nestedness, and connectance to remove the effect it might have on these structural network metrics. Indeed, some metrics might be sensitive to changes in network size; for example, nestedness tends to increase with larger networks, (Nielsen & Bascompte, 2007). We used multiple regression in the GLM models. For models with response variables in the form of proportions, we used the Beta distribution function, and for count response variables, the Poisson distribution function. We performed residual analysis to evaluate the error distribution. GLMs were also used to evaluate whether ectoparasites richness and parasitic indices varied along the latitudinal gradient. After performing all the analyses, we verified the existence of spatial autocorrelation in the residuals. All analyses were performed in the R environment (R Development Core Team, 2018), using the packages *glmmTMB*, *ncf*, *visreg*, and ggplot2 (Breheny & Burchett, 2017; Brooks et al., 2017; Nychka et al., 2002; Wickham, 2020).

## 3 RESULTS

**FIGURE 1:**
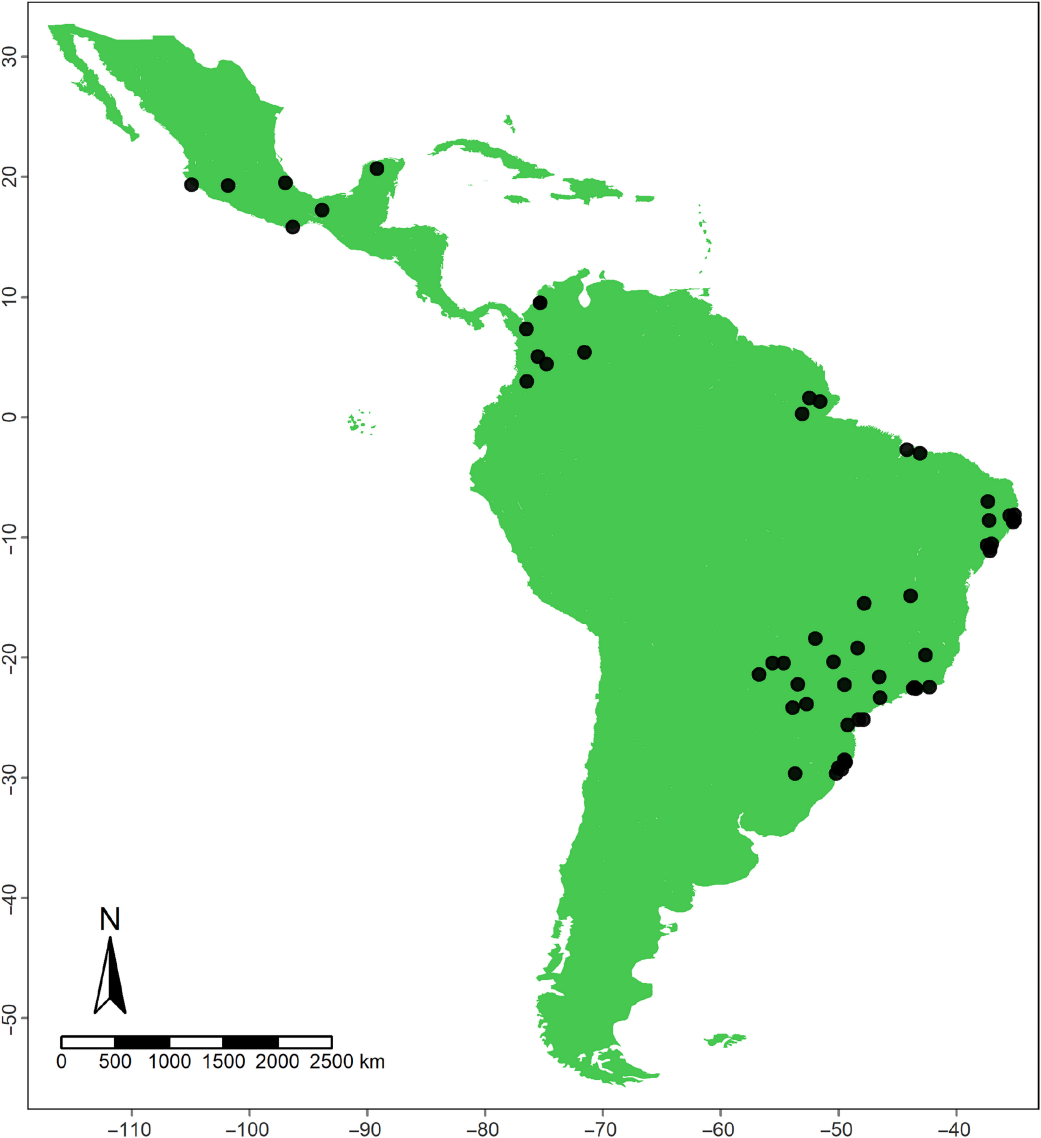
Sampling locations for the 48 studies retrieved in the bibliometric search, from which we generated 57 interaction networks between bats and ectoparasitic bat‐flies.

From the literature search, we obtained 49 research papers containing the necessary information for the analyses, from which we generated 57 interaction networks. The datasets were distributed from southern Mexico to southern Brazil, between latitudes 29° S and 19° N (Figure 1; Data S1).

**FIGURE 2:**
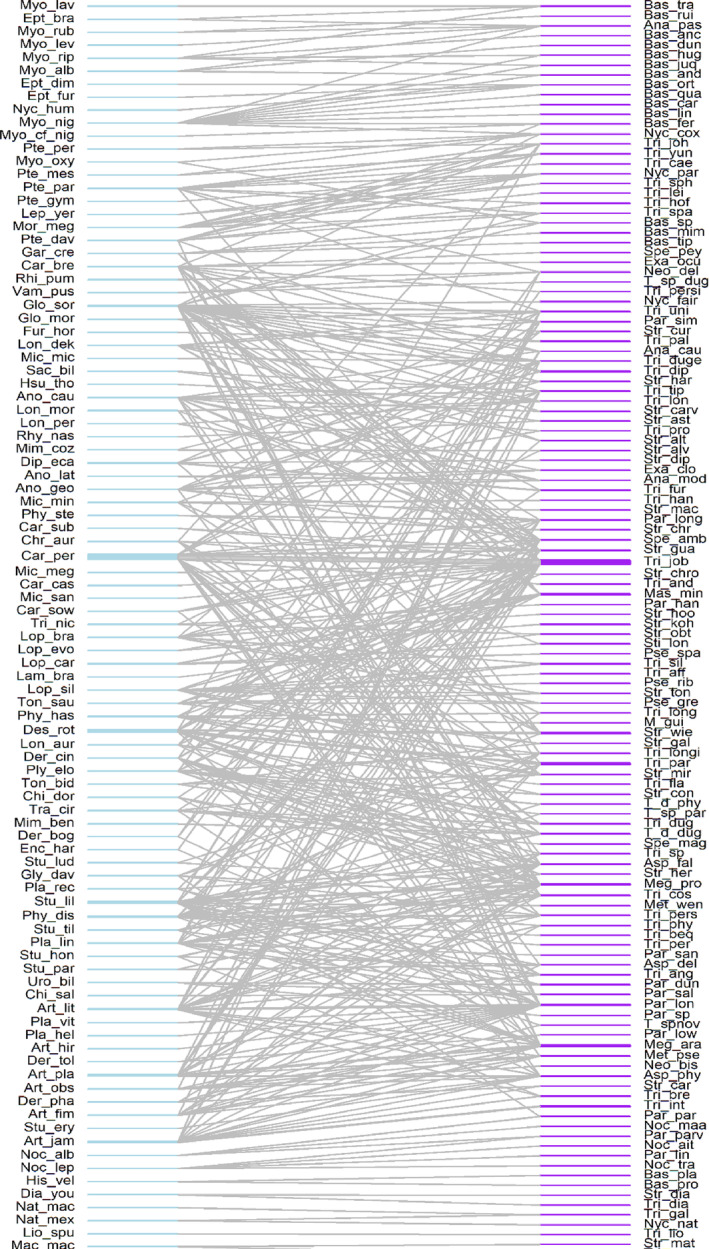
Interaction network with unified data for the entire Neotropical Region, between bats (Chiroptera) and their ectoparasitic flies (Streblidae and Nycteribiidae). Scientific names of both groups in code (Data S3).

We obtained data for 97 bat species, distributed in 43 genera and seven families, and 128 species of bat‐flies, 111 species and 20 genera of the Streblidae, and 17 species of the Nycteribiidae, all from the genus *Basilia* (Figure 2; Data S2). The bat species with the highest parasite richness was *Glossophaga soricina* (Pallas, 1766), with 28 species of ectoparasites, followed by *Carollia perspicillata* (Linnaeus, 1758), with 22 species of bat‐flies. Of all the bat species parasitized in this study, 29 were parasitized by solely one species of bat fly.

**FIGURE 3:**
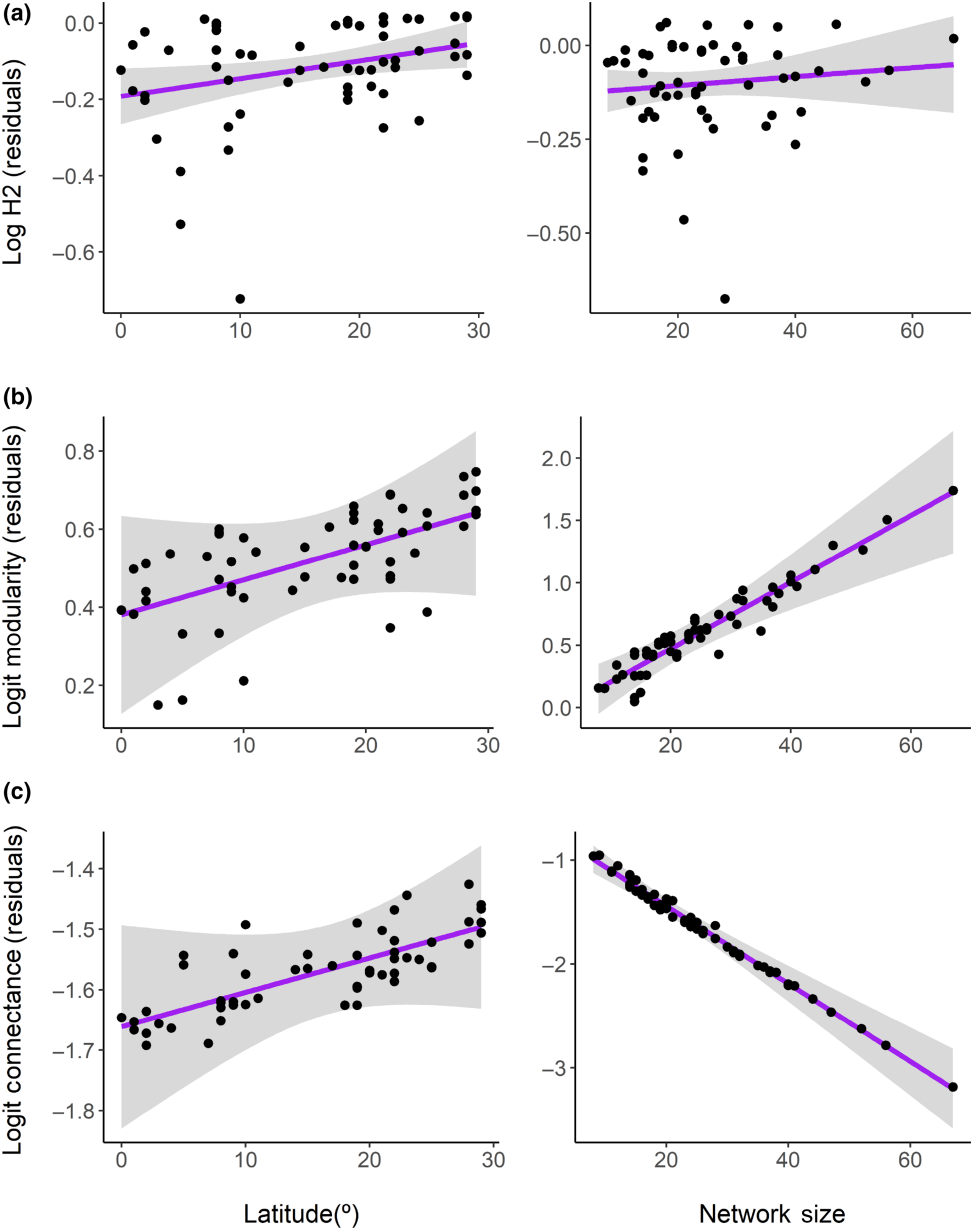
Variation of (a) specialization (H2), (b) modularity, and (c) connectance of networks between bats and their ectoparasitic flies in the Neotropical region in response to latitude and network size.

**FIGURE 4:**
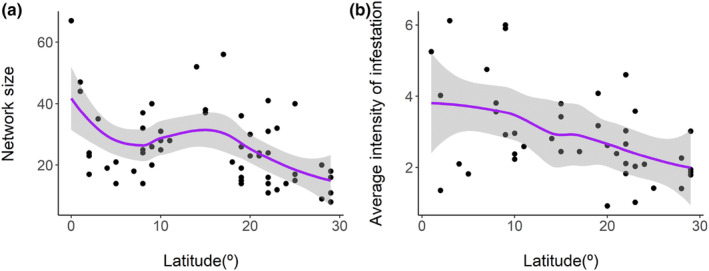
Variation in the size of the networks between bats and their ectoparasitic flies with latitude (a) and variation in the average intensity of infestation with latitude (b), in the Neotropical region.

The specialization index (H2) was high throughout the Neotropic, ranging from 0.45 to 1.00, with the vast majority (83%) above 0.80. H2 responded positively to latitude, that is as latitude increases, networks tended to be more specialized (*β* = 2.298; *p* = .025; *R* ^2^ = 0.089 – Figure 3). Modularity ranged from 0.28 to 0.88, responding positively to the size of the network (*β* = 4.860; *p* < .001; *R* ^2^ = 0.296 – Figure 3). Nestedness and connectance were generally low, ranging from 0.00 to 23.34 and 0.05 to 0.33, respectively. Connectance responded negatively to the size of the network (*β* = −8.889; *p* < .001; *R* ^2^ = 0.677 – Figure 3). Network size ranged from 8 to 67, also responding negatively to latitude (*β* = −7.253; *p* < .001; *R* ^2^ = 0.154 – Figure 4a).

**FIGURE 5:**
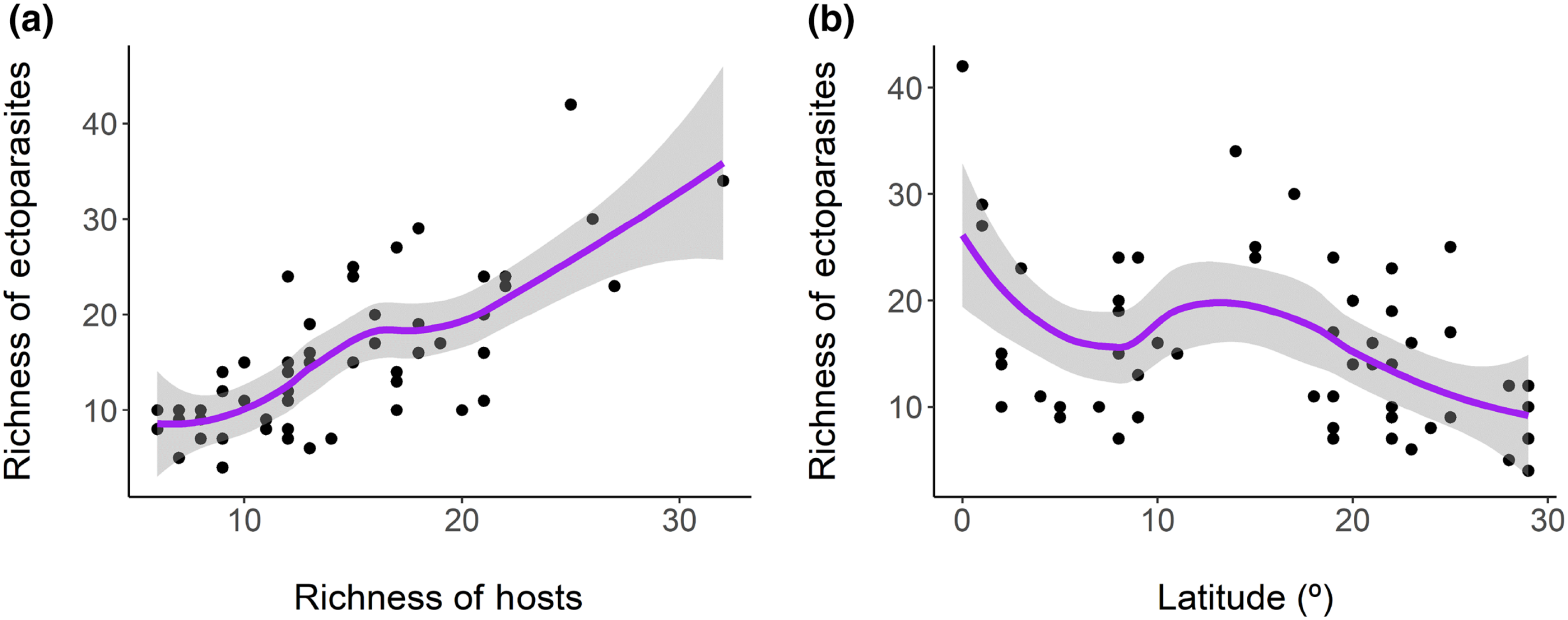
Variation in ectoparasite richness in relation to host richness (a) and latitudinal gradient (b), along the Neotropical region.

The richness of ectoparasites responded positively to the richness of their hosts (*F* = 30.21; *p* < .001; *R* ^2^ = 0.783 – Figure 5a), which indirectly influenced the opposite response for latitude (Figure 5b). Prevalence ranged from 20.12% to 84.30%, but did not respond to latitude, despite the mean infestation intensity has shown a negative relation with latitude (*F* = 11.84; *p* = .001; *R* ^2^ = 0.224), ranging from 0.92 to 6.12 (Figure 4b).

## 4 DISCUSSION

Here we present the first evaluation of the structuring of the interaction networks between bats and their ectoparasitic flies, as well as of parasitic indices, at a biogeographic scale along the Neotropical region. Although the number of studies on interactions between bats and their ectoparasitic flies has increased in recent years, the vast majority are still carried out at local or regional scales (e.g., Biz et al., 2021; Fagundes et al., 2017; Hiller et al., 2020; Salinas‐Ramos et al., 2018; Tlapaya‐Romero et al., 2021; Trujillo‐Pahua & Ibáñez‐Bernal, 2020). Fewer studies have provided data gathered on larger scales (but see Eriksson et al., 2019; Júnior et al., 2020; Saldaña‐Vázquez et al., 2019). Júnior et al. (2020) provide extremely relevant information on network structures for bats and ectoparasites on a global scale. Our study sought to bring new information about the interaction between the two groups on a biogeographic scale, adding information such as network specialization and parasite indices, and focusing specifically on the Neotropical region, where the Streblidae family presents its peak of diversity (Dick & Graciolli, 2013) mostly because it seems to preferably parasitize the Neotropical endemic Phyllostomidae (Taylor & Turttle, 2019).

Interaction patterns at the local scale (Durán et al., 2019; Fagundes et al., 2017; Hiller et al., 2021) may hide variations in the relationship between species, but when observed at a larger scale may reveal modulations, as we detected here. Knowing that parasites are highly specific and that their distribution is limited not only by the presence of their hosts but also by other environmental variables (Marshall, 1982), the latitudinal variation in the structuring of the ecological networks was expected. Indeed, not only the type of interaction affects network structure, but also those environmental factors of which latitude is only a proxy, such as temperature and humidity (Tlapaya‐Romero et al., 2021), as they vary on large scales, but remain reasonably stable on smaller scales (Alvares et al., 2013).

The latitudinal diversity gradient is a geographical pattern in the distribution of species, in which the closer to the tropics, the greater the diversity, and this occurs in a multiplicity of biological taxa (Allen & Gillooly, 2006). Some of the main hypotheses that seek to explain this general pattern of biodiversity are based on environmental and historical factors, such as: climate/productivity; environmental heterogeneity, seasonality and dispersion/history (Campo et al., 2008; Tello & Stevens, 2010). Thinking about this concept, latitude becomes a proxy for several environmental factors that vary along a gradient (Gouveia et al., 2012; Tello & Stevens, 2010). This general rule has exceptions in some groups (exceptions mostly known in some plant and bird families) but is marked in bats and their ectoparasitic flies (Biz et al., 2021; Durán et al., 2017; Ramos Pereira & Palmeirim, 2013). Indeed, we detected an indirect effect of latitude on network metrics essentially because latitude influences network size. Sites with greater species richness should harbor a greater number of ecological interactions, with the consequent increase in the size of the networks and, consequently, larger networks, that is those with a higher number of species, should be found at lower latitudes. Despite the increase in species richness toward the tropics, specialization remained high in lower latitudes, though slightly (but significantly) greater in higher latitudes. Such suggests that the traditionally known pattern in ecology regarding the latitudinal gradient in specialization—species tend to be more specialized in the tropics—is not as a rule, and the results can be mixed (Moles & Ollerton, 2016). Meta‐analysis studies have concluded that there is not much evidence for a general effect of latitude on niche breadth, and that biotic interactions are not always more specialized at lower latitudes (Vázquez & Stevens, 2004). This pattern in specialization possibly also results from the extremely high specificity (Marshall, 1982) shown by bat flies of the Streblidae and Nycteribiidae. In those families, 87% of the species are monoxenic, parasitizing a single bat species (Dick, 2007), and a few are found on congener hosts or on a second, non‐primary, host (Dick, 2007). At lower latitudes, there is overlap in the geographic distribution of host species of oligoxenous and polyxenous flies, which associate with congener and confamilial hosts (Dick, 2007; Graciolli & Dick, 2004; Taylor & Turttle, 2019), while at higher latitudes there is lower richness of hosts (Ramos Pereira & Palmeirim, 2013), making the interactions between ectoparasites and bats somewhat more restricted, that is, specialized.

The behavioral and biological attributes of bats and bat flies may influence the intensity of parasitic loads. Bat species aggregating in colonies with many individuals in more stable roosts tend to harbor a greater number and richness of ectoparasites (Patterson et al., 2007). The best explanation for this pattern is related to the reproductive biology of bat flies. The two families of bat flies present adenotrophic viviparity (Hagan, 1951), in which the female must leave the host to deposit a single third instar larva on the substrate of the roost (Marshall, 1982). Subsequently, this larva takes around 28 days to hatch into an adult fly, and then search for a potential host (Marshall, 1970). Thus, ectoparasite survival increases the longer the hosts stay in the roosts. Still, bat roosting ecology is extremely varied (Kunz & Lumsden, 2003) from species consistently forming colonies of thousands of individuals in permanent roosts, particularly caves, to species that roost alone or form small groups of a few individuals in ephemeral shelters, such as foliage (Kunz & Lumsden, 2003). Other species of bats show significant plasticity in the choice of roosts and in the solitary‐gregarious gradient, shaping roost occupancy patterns according to roost and food availability in a given area (Kunz & Lumsden, 2003).

Morphological aspects of the hosts may also play a role in increasing parasite loads, such as body size (Patterson et al., 2008). Body size, when compared between different species of bats, seems to have an influence only when the species under comparison have cave‐dwelling habits; host species that use more ephemeral roosts, even with larger body sizes, tend to have lower parasite indices when compared to smaller cave‐dwelling species (Patterson et al., 2008). This reveals the importance of roost ecology for the survival of bat flies. Larger species with greater plasticity in the choice of the roost may have distinct parasite loads along an environmental gradient (Kunz & Lumsden, 2003), eventually showing higher parasite loads in tropical regions, due to the higher availability of caves in these regions. Indeed bats that use caves opportunistically may have larger parasite load and richness when using caves, but may show a decrease in several parasitological parameters when they occupy other roost types. (Guimarães & Ferreira, 2014). However, such patterns have only been described for a few species, and only for mean abundance and mean number of species of ectoparasitic flies; patterns for most bat species and for parasite intensity are yet to be described (Patterson et al., 2008; Presley & Willing, 2008).

The ecological diversity of their hosts, associated with environmental changes, including in the landscape or climate, may lead to observable changes in the reproductive success of ectoparasitic bat‐flies (Reckardt & Kerth, 2007; Tlapaya‐Romero et al., 2021). Indeed, higher temperatures result in shorter incubation times for other groups of bat ectoparasites, increasing reproduction rates (Tlapaya‐Romero et al., 2021). The higher infestation intensity we found at lower latitudes may be explained by higher average temperatures along the year and by the more predictable regional climates overall. Larger, more stable, and permanent roosts are possibly also more available here (Cecav, 2020; Guimarães & Ferreira, 2014), and these can harbor higher host richness (Barros et al., 2021). In tropical regions, the formation of bat colonies with thousands of individuals is notable, especially in those species using underground roosts obligatorily or frequently. In the Neotropic, only *Tadarida brasiliensis* presents records of large colonies beyond the tropical region (Boero et al., 2020; Vargas‐Mena et al., 2018). De Oliveira et al. (2018) observed greater number of individuals in the Brazilian savanna, when compared to the Atlantic Forest, which extends to higher latitudes. Considering that the epidemiological modeling theory postulates that the transmission of parasites between hosts is influenced by population density (Poulin, 2004), the larger the host colony, the greater the probability of infection. A corollary of this theory is that parasite abundance will respond positively to host density (Arneberg et al., 1998). In conclusion, environmental changes along the latitudinal gradient will directly influence host abundance and, subsequently, also the parasitic infestation patterns.

Interaction networks indirectly respond to latitude in a pattern that is only perceptible on a large scale, a finding revealing the need for further macroecological studies to understand the factors behind the structure of those ecological interactions. Future studies on interaction patterns between bats and their ectoparasitic flies should consider the roosting ecology of the hosts and the latitudinal variation of environmental variables such as landscape, climate, and roost permanence and availability.

## AUTHOR CONTRIBUTIONS

**Luana S. Biz:** Conceptualization (equal); data curation (lead); formal analysis (lead); investigation (lead); methodology (lead); writing – original draft (lead); writing – review and editing (equal). **Vinicius A. G. Bastazini:** Conceptualization (equal); funding acquisition (equal); methodology (equal); supervision (supporting); writing – review and editing (equal). **Fernando Carvalho:** Writing – original draft (equal). **Maria João Ramos Pereira:** Conceptualization (equal); formal analysis (supporting); funding acquisition (equal); methodology (equal); resources (lead); supervision (lead); writing – original draft (supporting); writing – review and editing (equal).

## CONFLICT OF INTEREST STATEMENT

The authors declare no competing interests.

## Supporting information


Data S1

Data S2

Data S3

## Data Availability

The data are provided in the manuscript, or are presented in [Link], [Link], [Link].
