# Supplementary material for: Network and parasitological analyses reveal latitudinal gradient in bats‐ectoparasitic fly interactions across the Neotropic
Source: Ecol Evol. 2023 Sep 15;13(9):e10527. doi: 10.1002/ece3.10527 (PMC10502467; doi:10.1002/ece3.10527)
Supplement: Supplementary file 1 — Data S1 [file ECE3-13-e10527-s002.pdf]

Supplementary Data 1. List of 49 articles used to generate 53 interaction networks throughout the Neotropical Region.

References	Nº Network
AGUIAR, L. M. S., and Y. ANTONINI. Prevalence and intensity of Streblidae in bats from a Neotropical savanna region in Brazil. *Folia Parasitologica*, v. 63, p. 1-8, 2016.	1
ÁLVAREZ, J.R., OSORIO, C.G., AUTINO, A.G., DIAS, L.G. First records of ectoparasitic insects (Diptera: Hippoboscoidea) of bat in the department of Caldas, Colombia. *Papéis Avulsos de Zoologia*, v. 60, p. 1-9, 2020.	1
ASCUNTAR-OSNAS, O., MONTOYA-BUSTAMANTE, S., GONZÁLEZ-CHÁVEZ, B. Records of Streblidae (Diptera: Hippoboscoidea) in a tropical dry forest fragment in Colombia. *Biota Colombiana*, v. 21, p. 16-27, 2020.	1
AZEVEDO, A.A., LINARDI, P.M. Streblidae (Diptera) of phyllostomid bats from Minas Gerais, Brazil. *Memórias Instituto Oswaldo Cruz*, v. 97, p. 421-422, 2002.	1
BARBIER, E., and G. GRACIOLLI. 2016. Community of bat flies (Streblidae and Nycteribiidae) on bats in the Cerrado of Central-West Brazil: hosts, aggregation, prevalence, infestation intensity, and infracommunities. Studies on Neotropical Fauna and Environment, 51(3): 1–12.	1
BARBIER, E., GRACIOLLI, G., BERNARD, E. Structure and composition of Nycteribiidae and Streblidae flies on bats along na environmental gradient in northeastern Brazil. *Canadian Journal Zoology*, v. 97, p. 1-37, 2018.	3
BARBIER, E., PRATO-NETO, J.G., BERNARD, E. Records of bats flies (Diptera: Nycteribiidae and Streblidae) in the semi-arid Caatinga in the state of Paraíba, northeastern Brazil. *Neotropical Entomology*, v. 45, p. 740-745, 2016.	1
BARBIER, E., URBIETA, G.L., NUNES, H., BOMFIM, S.S., ROCHA, P.A. High specificity and aggregation, but low prevalence in bat-fly interactions in an environmental protection area in Brazil. *Acta Chiropterologica*, v. 21, p. 443-452, 2019.	1
BERTOLA, P. B., C. C. AIRES, S. E. FAVORITO, G. GRACIOLLI, M. AMAKU, and R. PINTO-da-ROCHA. 2005. Bat flies (Diptera: Streblidae, Nycteribiidae) parasitic on bats (Mammalia: Chiroptera) at Parque Estadual da Cantareira, São Paulo, Brazil: parasitism rates and host-parasite associations. Memórias do Instituto Oswaldo Cruz, v. 100, p. 25–32.	1
BEZERRA, R. H. S., P. F. VASCONCELOS, and A. BOCCHIGLIERI. 2016. Ectoparasites of bats (Mammalia: Chiroptera) in Atlantic forest fragments in northeastern Brazil. Parasitology Research, 115: 3759–3765.	1
BEZERRA, R.H.S., BOCCHIGLIERI, A. Association of ectoparasites (Diptera and Acari) on bats (Mammalia) in a restinga habitat in northeastern Brazil. *Parasitology Research*, v. 117, p. 3413-3420, 2018.	1
BIZ, L.S., CASCAES, M.F., LUCIANO, B.F.L., PREUSS, G., BÔLLA, D.A.S., GRACIOLLI, G., CARVALHO, F. Parasitic interactions between bats (Mammalia: Chiroptera) and flies (Insecta: Diptera) in the intersection area of temperate and tropical climates in Brazil. *Studies on Neotropical Fauna and Environment*, v. 2021.	1

CALONGE-CAMARGO, B., PÉREZ-TORREZ, J. Ectoparasites (Polyctenidae, Streblidae, Nycteribiidae) of bats (Mammalia: Chiroptera) from the Caribbean region of Colombia. *Therya*, v. 9, p. 171-178, 2018.	1
CAMILOTTI, V.L., GRACIOLLI, G., WEBER, M.M., ARRUDA, J.L.S., CÁCERES, N.C. Bat flies from the deciduous Atlantic Florest in southern Brazil: host-parasite relationships and parasitismo rates. *Acta Parasitologica*, v. 55, p. 194-200, 2010.	1
CUXIM-KOYOC, A., E. REYES-NOVELO, J. B. MORALES-MALACARA, B. BOLÍVAR-CIMÉ, and J. LABORDE. 2015. Streblidae (Diptera: Hippoboscoidea) from Yucatan and Updated species List for Mexico. Journal of Medical Entomology, 52(5): 947–961.	1
CUXIM-KOYOC, A., REYES-NOVELO, E., MACSWINEY, C.M., AGUILAR-RODRIGUÉZ, P.A. Nuevos registros de Streblidae (Diptera: Hippoboscoidea) para México. *Revista Colombiana de Entomología*, v. 42, p. 192-196, 2016.	1
CUXIM-KOYOC, A., REYES-NOVELO, E., MACSWINEY, C.M., PECH-CANCHÉ, J.M. Moscas ectoparásitas de murciélagos (Diptera:Streblidae y Nycteribiidae) del valle de Uxpanapa, Veracruz, México. *Revista Mexicana de Biodiversidad*, v. 89, p. 1074-1088, 2018.	1
DORNELLES, G. D. P., and G. GRACIOLLI. 2017. Streblid bat flies on phyllostomid bats from na island off the coast of São Paulo, Brazil. Papéis Avulsos de Zoologia, 57(4): 31–36.	1
DORNELLES, G.P.D., GRACIOLLI, G., ODON, A., BORDIGNON, M.O. Infracommunities of Streblidae and Nycteribiidae (Diptera) on bats in ecotone area between Cerrado and Atlantic Florest in the state of Mato Grosso do Sul. *Iheringia*, v. 107, p. 1-5, 2017.	1
DURÁN, A. A., D. M. Á. GARCÍA, and G. GRACIOLLI. 2017. Ectoparasitic flies (Diptera, Streblidae) on bats (Mammalia, Chiroptera) in a dry tropical forest in the Northern Colombia. Papéis Avulsos de Zoologia, 57(8): 105–111.	1
DURÁN, A.A., SALDAÑA-VÁZQUEZ, R.A., GRACIOLLI, G., PEINADO, L.C. Specialization ond modularity of a bat antagonistic ecological network in a dry tropical forest in northern Colombia. *Acta Chiropterologica*, v. 20, p. 503-510, 2018.	1
ERIKSSON, A., G. GRACIOLLI, and E. FISCHER. 2011. Bat flies on phyllostomid hosts in the Cerrado region: component community, prevalence and intensity of parasitism. Memórias do Instituto Oswaldo Cruz, 106(3): 274–278	1
FRANÇA, D. S., S. N. PEREIRA, A. C. S. MAAS, M. A. MARTINS, D. P. BOLZAN, I. P. LIMA, D. DIAS, and A. L. PERACCHI. 2013. Ectoparasites flies (Diptera, Streblidae) of bats (Chiroptera, Phyllostomidae) in an Atlantic Florest area, southeastern Brazil. Brazilian Journal of Biology, 73(4): 847–854.	1
GRACIOLLI, G. RUI, A.M. Streblidae (Diptera, Hippoboscoidea) em morcegos (Chiroptera, Phyllostomidae) no nordeste do Rio Grande do Sul, Brasil. *Iheringia*, v. 90, p. 85-92, 2001.	1
GRACIOLLI, G., BIANCONI, G.V. Moscas ectoparasitas (Diptera, Streblidae e Nycteribiidae) em morcegos (Mammalia, Chiroptera) em área de floresta com araucária no estado do Paraná, sul do Brasil. *Revista Brasileira de Zoologia*, v. 24, p. 246-249, 2007.	1
GRACIOLLI, G., LINARDI, P.M. Some Streblidae and Nycteribiidae (Diptera: Hippoboscoidea) from Maracá Island, Roraima, Brazil. *Memórias Instituto Oswaldo Cruz*, v. 97, p. 139-141, 2002.	1

GRACIOLLI, G., M. ZORTÉA, and L. F. A. C. CARVALHO. 2010. Bat flies (Diptera, Streblidae and Nycteribiidae) in a Cerrado area of Goiás State, Brazil. Revista Brasileira de Entomologia, 54(3): 511–514.	1
GRACIOLLI, G., PASSOS, F.C., PEDRO, W.A., LIM, B.K. Moscas ectoparasitas (Diptera, Streblidae) de morcegos filostomídeos (Mammalia, Chiroptera) na Estação Ecológica dos Caetetus, São Paulo, Brasil. *Revista Brasileira de Zoologia*, v. 23, p. 298-299, 2006.	1
HRYCYNA, G., MARTINS, A.C.M., GRACIOLLI, G. Infracommunities of bat flies (Diptera: Streblidae and Nycteribiidae) of bats (Mammalia: Chiroptera) in three conservation units in the state of Amapá, Brazil. *Biota Neotropica*, v. 19, p. 1-9, 2019.	3
JÚNIOR, L.F.M., MENEZES, A.C.D.P., SANTOS, D.M.C., PERACCHI, A.L. Ectoparasitic flies (Diptera: Streblidae) on bats (Mammalia: Chiroptera) from a Private Natural Heritage Reserve in southeastern Brazil. *Papéis Avulsos de Zoologia*, v. 61, p. 1-6, 2021.	1
KOMENO, C.A., LINHARES, A.X. Batflies parasitic on some phyllostomid bats in southeastern Brazil: parasitism rates and host-parasite relationships. *Memórias Instituto Oswaldo Cruz*, v. 94, p. 151-156, 1999.	1
LIÉVANO-ROMERO, K.S., RODRÍGUEZ-POSADA, M.E., CORTÉS-VECINO, J.A. Nuevos registros de ectoparasitos de murciélagos em sabanas inundables de la orinoquía Colombiana. *Mastozoologia Neotropical*, v. 26, p. 377-389, 2019.	1
LOURENÇO, E. C., P. M. P. PATRÍCIO, M. C. PINHEIRO, R. M. DIAS, and K. M. FAMADAS. 2014. Streblidae (Diptera) on bats (Chiroptera) in an area of Atlantic Florest, state of Rio de Janeiro. Brazilian Journal of Veterinary Parasitology, 23(2): 164–170.	1
LOURENÇO, E.C., GOMES, L.A.C., VIANA, A.O., FAMADAS, K.M. Co-occurrence of ectoparasites (Insecta and Arachnida) on bats (Chiroptera) in an Atlantic Forest Remnant, southeastern Brazil. *Acta Parasitologica*, v. 65, p. 750-759, 2020.	1
MORAS, L. M., L. F. O. BERNARDI, G. GRACIOLLI, and R. GREGORIN. 2013. Bat flies (Diptera: Streblidae, Nycteribiidae) and mites (Acari) associated with bats (Mammalia: Chiroptera) in a high-altitude region in southern Minas Gerais, Brazil. Acta Parasitologica, 58(4): 556–563.	1
RIBAS, M.R., BATISTA, S.C., ARANHA, J.M.R. Occurrence and infestation rates of Streblidae (Diptera, Hippoboscoidea) on bats (Mammalia, Chiroptera) in a semideciduous seasonal forest fragment in western Paraná, Brazil. *Iheringia*, v. 110, p. 1-8, 2020.	1
RUI, A.M., GRACIOLLI, G. Moscas ectoparasitas (Diptera, Streblidae) de morcegos (Chiroptera, Phyllostomidae) no sul do Brasil: associações hospedeiros-parasitos e taxas de infestação. *Revista Brasileira de Zoologia*, v. 22, p. 438-445, 2005.	1
SANTOS, C. L. C., P. A. DIAS, F. S. RODRIGUES, K. S. LOBATO, L. C. ROSA, T. G. OLIVEIRA, and J. M. M. REBÊLO. 2009. Moscas Ectoparasitas (Diptera: Streblidae) de Morcegos (Mammalia: Chiroptera) do Município de São Luís, MA: Taxas de Infestação e Associações Parasito-Hospedeiro. Neotropical Entomology, 38(5): 595-601.	1
SANTOS, C.L.C., PEREIRA, A.C.N., BASTOS, V.J.C., GRACIOLLI, G., REBÊLO, J.M.M. Parasitismo of ectoparasitic flies on bats in the northern brazilian cerrado. *Acta Parasitologica*, v. 58, p. 207-214, 2013.	1

SILVA, J. R. R., and H. O. FILHO. 2011. Dípteros ectoparasitas (Insecta, Diptera) em morcegos (Chiroptera, Mammalia) na Reserva Biológica das Perobas Paraná, Brasil. Iheringia, Série Zoologia, 101(3): 220–224.	1
SOARES, F. A. M., G. GRACIOLLI, C. E. B. P. RIBEIRO, R. S. BANDEIRA, J. A. T. MORENO, and S. F. FERRARI. 2016. Bat (Mammalia: Chiroptera) diversity in an area of mangrove forest in southern Pernambuco, Brazil, with a new species record and notes on ectoparasites (Diptera: Streblidae). Papéis Avulsos de Zoologia, 56(6): 63–68.	1
SOARES, F. A. M., G. GRACIOLLI, D. M. C. ALCÂNTARA, C. E. B. P. RIBEIRO, G. C. VALENÇA, and S. F. FERRARI. 2013. Bat flies (Diptera: Streblidae) ectoparasites of bats at na Atlantic Rainforest site in northeastern Brazil. Biota Neotropica, 13(2): 1–5.	1
SOARES, F.A.M., ROCHA, P.A., MIKALAUSKAS, J.S., GRACIOLLI, G., FERRARI, S.F. Ectoparasitic bat flies (Diptera, Streblidae) of bats (Chiroptera, Mammalia) from Mata do Junco Wildlife Refuge,Sergipe, northeastern Brazil. *Oecologia Australis*, v. 21, p. 385-395, 2017.	1
TARQUINO-CARBONELL, A. P., K. A. GUTIÉRREZ-DÍAZ, E. Y. GALINDO-ESPINOSA, G. REINOSO-FLÓREZ, S. SOLARI, and R. GUERRERO. 2015. Ectoparasites associated with bats in northeastern Tolima, Colombia. Mastozoología Neotropical, 22(2): 349–358.	1
TLAPAYA-ROMERO, L., IBÁÑEZ-BERNAL, S., SANTOS-MORENO, A. New records of bat flies (Diptera: Streblidae) in Oaxaca, Mexico. *Revista Mexicana de Biodiversidad*, v. 90, p. 1-16, 2019.	1
TRUJILLO-PAHUA, L., IBÁÑEZ-BERNAL, S. New geographical records of bat flies (Diptera: Streblidae) associated with phyllostomid bats (Chiroptera: Phyllostomidae) in the west highlands of México. *Journal of Medical Entomology*, v. 56, p. 18-28, 2019.	1
URBIETA, G.L., TORRES, J.M., ANJOS, E.A.C., CARVALHO, C.M.E., GRACIOLLI, G. Parasitismo of bat flies (Nycteribiidae and Streblidae) on bats in urban environments: lower prevalence, infracommunities, and specificity. *Acta Chiropterologica*, v. 20, p. 511-518, 2018.	1
VASCONCELOS, P.F., FALCÃO, L.A.D., GRACIOLLI, G., BORGES, M.A.Z. Parasite-host interactions of bat flies (Diptera: Hippoboscoidea) in Brazilian tropical dry forests. *Parasitology Research*, v. 115, p. 367-377, 2016.	1
ZARAZÚA-CARBAJAL, M., R. A. SALDAÑA-VÁZQUEZ, C. A. SANDOVAL-RUIZ, K. E. STONER, and J. BENITEZ-MALVIDO. 2016. The specificity of host-bat fly interaction networks across vegetation and seasonal variation. Parasitology Research, 115(10): 4037–4044.	1
