# Supplementary material for: Network and parasitological analyses reveal latitudinal gradient in bats‐ectoparasitic fly interactions across the Neotropic
Source: Ecol Evol. 2023 Sep 15;13(9):e10527. doi: 10.1002/ece3.10527 (PMC10502467; doi:10.1002/ece3.10527)
Supplement: Supplementary file 2 — Data S2 [file ECE3-13-e10527-s001.pdf]

Supplementary Data 2. Associations between bat species and ectoparasite species found in 57 interaction networks distrubuted throughout the Neotropical region.

Bat species	Ectoparasite species
Anoura caudifer (É. Geoffroy, 1818)	*Trichobius tiptoni* Wenzel, 1976
	Exastinion clovisi (Pessôa & Guimarães, 1937)
	Anastrebla caudiferae Wenzel, 1976
	Anastrebla modestini Wenzel, 1966
	Trichobius joblingi Wenzel, 1966
	Streblaguajiro (García & Casal, 1965)
	Strebla carvalhoi Graciolli, 2003
	Trichobius propinquus Wenzel, 1976
	Strebla harderi Wenzel, 1976
	Paratrichobius longicrus (Miranda Ribeiro, 1907)
	Megistopoda proxima (Séguy, 1926)
	Strebla wiedemanni Kolenati, 1856
Artibeus jamaicenses Leach, 1821	*Aspidoptera phyllostomatis* (Perty, 1833)
	Trichobius intermedius Peterson & Hürka, 1974
	Megistopoda aranea (Coquillett, 1899)
	Metelasmus pseudopterus Coquillett, 1907
	Aspidoptera delatorrei Wenzel, 1966
	P. longicrus
	S. guajiro
	T. joblingi
	Trichobius parasiticus Gervais, 1844
	Trichobius uniformis Curran, 1935
	Speiseria ambigua Kessel, 1925
	M. proxima
	Trichobius brennani Wenzel, 1966
Artibeus fimbriatus Gray, 1838	*P. longicrus*
	M. aranea
	M. pseudopterus
	Aspidoptera falcata Wenzel, 1976
	A. phyllostomatis
	T. tiptoni
	M. proxima
	T. spnov
	Trichobius phyllostomae Kessel, 1925
Anoura geoffroyi Gray, 1838	*E. clovisi*
	S. harderi
	A. modestini
	Paraeuctenodes longipes Pessôa & Guimarães, 1937
	T. propinquus
	Streblacurvata Wenzel, 1976
	S. ambigua

	T. tiptoni<br>*Aspidoptera delatorrei* Wenzel, 1966
Artibeus hirsutus K.Andersen, 1906	*P. longicrus*
Artibeus lituratus (Olfers, 1818)	*P. longicrus*<br>*M. aranea*<br>*T. joblingi*<br>*A. phyllostomatis*<br>*M. proxima*<br>*M. pseudopterus*<br>*A. falcata*<br>*Trichobius angulatus* Wenzel, 1976<br>*Trichobius sp.*<br>*T. uniformis*<br>*Trichobioides perspicillatus* (Pessôa & Galvão, 1937)<br>*Trichobius costalimai* Guimarães, 1938<br>*Trichobius longipes* (Rudow, 1871)<br>*T. tiptoni*<br>*T. brennani*<br>*T. phyllostomae*<br>*S. guajiro*
Artibeus obscurus (Schinz, 1821)	*M. aranea*<br>*M. pseudopterus*<br>*A. phyllostomatis*<br>*P. longicrus*<br>*Trichobius dugesii* Townsend, 1891<br>*Neotrichobius bisetosus* Wenzel, 1976
Dermanura phaeotis Miller, 1902	*Paratrichobius lowei* Wenzel, 1966<br>*P. longicrus*<br>*M. proxima*
Artibeus planirostris Spix, 1823	*M. aranea*<br>*A. phyllostomatis*<br>*T. joblingi*<br>*M. pseudopterus*<br>*A. falcata*<br>*P. longicrus*<br>*S. ambigua*<br>*S. guajiro*<br>*T. dugesii*<br>*T. longipes*<br>*T. parasiticus*<br>*Trichobius sp.*<br>*T. costalimai*
Dermanura tolteca (Saussure 1860)	*P. lowei*
Chrotopterus auritus (Peters, 1856)	*Trichobius dugesioides dugesioides* Wenzel, 1966<br>*Streblachrotopteri* Wenzel, 1976<br>*Trichobius dugesioides* Wenzel, 1966

	Streblamirabilis (Waterhouse, 1879)
	T. longipes
	Trichobius johnsonae Wenzel, 1966
	S. guajiro
Carollia brevicauda (Schinz, 1821)	*Speiseria peytonae* Wenzel, 1976
	T. joblingi
	Mastoptera minuta (Costa Lima, 1921)
	S. guajiro
	S. ambigua
	Trichobius persimilis Wenzel, 1976
	S. wiedemanni
	P. longicrus
	T. longipes
	T. tiptoni
	Paraeuctenodes similis Wenzel, 1976
	A. caudiferae
	Exastinion oculatum Wenzel, 1976
	Basilia ferrisi Schuurmans Stekhoven, 1931
Carollia castanea H.Allen, 1890	*T. joblingi*
Chiroderma doriae Thomas, 1891	*T. joblingi*
	S. guajiro
	A. falcata
	M. proxima
	T. angulatus
Carollia perspicillata (Linnaeus, 1758)	*T. joblingi*
	S. guajiro
	T. uniformis
	S. ambigua
	P. similis
	A. falcata
	M. aranea
	M. proxima
	T. tiptoni
	S. mirabilis
	P. longipes
	P. longicrus
	Trichobius anducei Guerrero, 1998
	S. wiedemanni
	T. dugesioides
	T. dugesii
	A. phyllostomatis
	Trichobioides perspicillatus (Pessôa & Galvão, 1937)
	T. costalimai
	T. parasiticus
	T. dugesioides dugesioides
	M. pseudopterus

Chiroderma salvini Dobson, 187	*Paratrichobius salvini* Wenzel, 1966
Carollia sowelli Baker, Solari & Hoffmann, 2002	*M. proxima*
	T. joblingi
	S. ambigua
	S. guajiro
Carollia subrufa (Hahn, 1905)	*T. joblingi*
Dermanura bogotensis (K.Andersen, 1906)	*P. longicrus*
	T. tiptoni
	P. similis
Artibeus cinereus (Gervais, 1856)	*Neotrichobius delicatus* Machado-Allison, 1966
	M. aranea
	T. joblingi
	S. ambigua
	Trichobius_sp
	T. perspicillatus
	A. falcata
	M. proxima
	T. costalimai
	Strebla hertigi Wenzel, 1966
	T. angulatus
Diphylla ecaudata Spix, 1823	*Strebladiphyllae* Wenzel, 1966
	Trichobius diphyllae Wenzel, 1966
	Trichobius furmani Wenzel, 1966
	T. uniformis
	S. mirabilis
	T. parasiticus
Desmodus rotundus (E. Geoffroy, 1810)	*S. wiedemanni*
	T. parasiticus
	S. wiedemanni
	T. joblingi
	T. dugesioides
	A. phyllostomatis
	S. mirabilis
	T. furmani
	S. ambigua
	A. falcata
	M. proxima
	S. hertigi
	T. dugesioides dugesioides
	T. johnsonae
Dermanura tolteca (Saussure, 1860)	*P. longicrus*
	M. proxima
Diaemus youngii (Jentink, 1893)	*Strebla diaemi* Wenzel, 1966
	Trichobius diaemi Wenzel, 1976
Eptesicus brasiliensis (Demarest 1819)	*Anatrichobius passosi* Graciolli, 2003
	Basilia travassosi Guimarães, 1938

Eptesicus diminutus Osgood, 1915	*Basilia ortizi* Machado-Allison, 1963
Eptesicus furinalis (d'Orbigny, 1847)	*B. ortizi*
Enchisthenes hartii (Thomas, 1892)	*P. longicrus*<br>*A. caudiferae*
Furipterus horrens (F. Cuvier, 1828)	*Trichobius pallidus* (Curran, 1934)
Glyphonycteris daviesi (Hill, 1964)	*T. perspicillatus*<br>*Paradyschiria parvuloides* Wenzel, 1966<br>*S. wiedemanni*<br>*T. costalimai*<br>*T. parasiticus*
Glossophaga morenoi Martínez & Villa-R., 1938	*T. dugesii*<br>*T. uniformis*
Glossophaga soricina (Pallas, 1766)	*T. dugesii*<br>*P. longipes*<br>*Trichobius lonchophyllae* Wenzel, 1966<br>*A. caudiferae*<br>*M. minutaa*<br>*S. curvata*<br>*S. ambigua*<br>*A. falcata*<br>*T. uniformis*<br>*T. joblingi*<br>*T. costalimai*<br>*S. carvalhoi*<br>*S. guajiro*<br>*T. furmani*<br>*P. longicrus*<br>*Strebla altmani* Wenzel, 1966<br>*S. harderi*<br>*P. similis*<br>*T. parasiticus*<br>*T. tiptoni*<br>*E. oculatum*<br>*Basilia sp.*<br>*M. aranea*<br>*Nycterophilia coxata* Ferris, 1916<br>*T. diphyllae*<br>*T. pallidus*<br>*Trichobius sparsus* Kessel, 1925<br>*Nycterophilia fairchildi* Wenzel, 1966
Hsunycteris thomasi J.A. Allen, 1904	*T. lonchophyllae*
Histiotus velatus (I. Geoffroy, 1824)	*Basilia plaumanni* Scott, 1940<br>*Basilia producta* Maa, 1968
Lonchorhina aurita Tomes, 1863	*Trichobius flagellatus* Wenzel, 1976<br>*S. altmani*<br>*T. dugesii*

	T. dugesioides<br>*T. perspicillatus*<br>*S. chrotopteri*
Lampronycteris brachyotis (Dobson, 1879)	*Strebla obtusa* Wenzel, 1976
Lophostoma brasiliense (Peters, 1866)	*M. minuta*<br>*T. longipes*<br>*Strebla hoogstraali* Wenzel, 1966<br>*Trichobius silvicolae* Wenzel, 1976<br>*T. joblingi*<br>*S. curvata*<br>*Streblatonatiae* (Kessel, 1924)<br>*Pseudostrebla greenwelli* Wenzel, 1966
Lophostoma carrikeri (J. A. Allen, 1910)	*Stizostrebla longirostris* Jobling, 1939<br>*M. minuta*<br>*Pseudostrebla sparsisetis* Wenzel, 1966
Lonchophylla dekeyseri Taddei, Vizotto, & Sazima, 1983	*T. dugesii*<br>*T. lonchophyllae*<br>*P. similis*<br>*T. uniformis*<br>*S. ambigua*<br>*S. guajiro*<br>*S. altmani*
Lophostoma evotis (Davis & Carter, 1978)	*M. minuta*<br>*Strebla kohlsi* Wenzel, 1966
Lonchophylla mordax Thomas, 1903	*Trichobius sp.*<br>*S. ambigua*<br>*T. lonchophyllae*
Lonchophylla peracchii Dias, Esbérard & Moratelli, 2013	*A. modestini*<br>*T. lonchophyllae*
Lophostoma silvicola d'Orbigny, 1836	*Trichobius affinis* Wenzel, 1976<br>*M. minuta*<br>*S. tonatiae*<br>*T. silvicolae*<br>*P. greenwelli*<br>*Pseudostrebla ribeiroi* Costa Lima, 1921<br>*S. mirabilis*<br>*Strebla galindoi* Wenzel, 1966<br>*T. parasiticus*
Lionycteris spurrelli Thomas, 1913	*Trichobius lionycteridis* Wenzel, 1966
Leptonycteris yerbabuenae Martínez and Villa-R., 1940	*Trichobius sphaeronotus* Jobling, 1939<br>*Nycterophilia parnelli* Wenzel, 1966
Myotis albescens (É. Geoffroy, 1806)	*Basilia andersoni* Peterson & Maa, 1970<br>*A. passosi*<br>*Basilia dunni* Curran, 1935
Mimon bennettii (Gray, 1838)	*T. dugesioides dugesioides*
Myotis nigricans (Schinz, 1821)	*B. andersoni*

	Basilia juquiensis Guimarães, 1946<br>*A. passosi*<br>*Basilia carteri* Scott, 1936<br>*B. ortizi*<br>*Anatrichobius scorzai* Wenzel, 1966<br>*B. ferrisi*<br>*Basilia hughscotti* Guimarães, 1946<br>*Basilia lindolphoi* Graciolli, 2001
Mimon cozumelae Goldman, 1914	*T. dugesii*<br>*Strebla alvarezi* Wenzel, 1966
Gardnerycteris crenulatum (É. Geoffroy, 1803)	*Basilia tiptoni* Guimarães, 1966<br>*Basilia mimoni* Theodor & Peterson, 1964<br>*T. joblingi*<br>*M. minuta*<br>*Basilia sp.*
Myotis keaysi J.A.Allen, 1914	*Trichobius hirsutulus* Bequaert, 1933
Myotis lavali Moratelli, Peracchi, Dias & Oliveira, 2011	*B. travassosi*
Myotis levis (I.Geoffroy, 1824)	*A. passosi*
Machophyllum macrophyllum (Schinz, 1821)	*Strebla matsoni* Wenzel, 1976<br>*Trichobius macrophylli* Wenzel, 1966
Mormoops megalophylla (Peters, 1864)	*T. johnsonae*<br>*T. sparsus*<br>*T. sphareonotus*<br>*N. parnelli*<br>*Trichobius yunkeri* Wenzel, 1966<br>*Trichobius leionotus* Wenzel, 1976
Micronycteris megalotis (Gray, 1842)	*T. joblingi*
Micronycteris microtis Miller, 1898	*T. dugesii*
Micronycteris minuta (Gervais, 1856)	*S. longirostris*<br>*Streblamachadoi* Wenzel, 1966<br>*Trichobius handleyi* Wenzel, 1976<br>*T. dugesii*<br>*S. wiedemannii*<br>*T. joblingi*
Trinycteris nicefori Sanborn, 1949	*S. alvarezi*<br>*S. obtusa*<br>*Parastrebla handleyi* Wenzel, 1966
Myotis oxyotus (Peters 1867)	*A. scorzai*<br>*Basilia sp.*
Myotis riparius Handley, 1960	*B. ferrisi*<br>*B. travassosi*<br>*Basilia anceps* Guimarães & d'Andretta, 1956<br>*B. juquiensis*<br>*B. hughscotti*
Myotis ruber (E.Geoffroy, 1806)	*A. passosi*<br>*Basilia ruiae* Graciolli, 2003

Micronycteris sanborni Simmons, 1996	*T. joblingi*
Myotis volans (H. Allen), 1866	*A. scorzai*
Noctilio albiventris Desmarest, 1818	*Paradyschiria parvula* Falcoz, 1931<br>*Noctiliostrebla maai* Wenzel, 1966<br>*P. parvuloides*
Nycticeius humeralis (Rafinesque, 1818)	*B. ortizi*
Noctilio leporinus (Linnaeus, 1758)	*Noctiliostrebla aitkeni* Wenzel, 1966<br>*Paradyschiria lineata* Kessel, 1925<br>*P. parvula*<br>*Noctiliostrebla traubi* Wenzel, 1966
Natalus macrourus (Gervais, 1856)	*Trichobius galei* Wenzel, 1966
Natalus mexicanus Miller, 1902	*Nycterophilia natali* Wenzel, 1966<br>*T. galei*
Pteronotus davyi Gray, 1838	*M. aranea*<br>*T. yunkeri*<br>*T. johnsonae*<br>*T. sparsus*<br>*Trichobius hoffmannae* Guerrero & Morales-Malacara, 1996<br>*T. diphyllae*<br>*N. parnelli*
Phyllostomus discolor Wagner, 1843	*S. hertigi*<br>*T. costalimai*<br>*Trichobius dugesioides phyllostomus* Guerrero, 1998<br>*T. perspicillatus*<br>*T. longipes*<br>*T. parasiticus*<br>*Trichobius bequaerti* Wenzel, 1966<br>*M. aranea*<br>*T. dugesioides dugesioides*<br>*T. joblingi*
Phyllostomus elongatus (É. Geoffroy, 1810)	*T. joblingi*<br>*T. dugesioides phyllostomus*<br>*Strebla consocia* Wenzel, 1966<br>*T. persimilis*<br>*T. longipes*<br>*T. costalimai*<br>*T. parasiticus*<br>*M. minuta*<br>*M. proxima*<br>*T. dugesioides*
Pteronotus gymnonotus Natterer, 1843	*N. parnelli*
Phyllostomus hastatus (Pallas, 1767)	*T. longipes*<br>*M. minutaa*<br>*S. mirabilis*<br>*S. consocia*<br>*Mastoptera guimaraesi* Wenzel, 1966

Platyrrhinus helleri (Petters, 1866)	*P. longicrus*
Platyrrhinus lineatus (E. Geoffroy, 1810)	*P. longicrus*
	T. angulatus
	M. proxima
	T. joblingi
	Paratrichobius sanchezi Wenzel, 1966
	N. delicatus
	T. perspicillatus
	T. costalimai
	S. hertigi
	T. longipes
	P. salvini
	A. falcata
	A. caudiferae
Pteronotus mesoamericanus Smith, 1972	*N. coxata*
	T. yunkeri
Pteronotus parnellii (Gray, 1843)	*T. johnsonae*
	Trichobius caecus Edwards, 1918
	N. parnelli
	M. minuta
	N. delicatus
	T. sparsus
	T. yunkeri
	T. hoffmannae
	T. uniformis
Pteronotus personatus (Wagner, 1843)	*T. johnsonae*
	T. yunkeri
	N. coxata
Platyrrhinus recifinus (Thomas, 1901)	*P. longicrus*
	S. ambigua
	A. falcata
	Paratrichobius dunni (Curran, 1935)
Phylloderma stenops Peters, 1865	*Strebla christinae* Wenzel, 1966
Platyrrhinus vittatus (Peters, 1860)	*P. longicrus*
Rhynchonycteris naso (Wied-Neuwied, 1820)	*Strebla asternalis* Wenzel, 1976
Rhinophylla pumilio Peters, 1865	*N. delicatus*
Saccopteryx bilineata (Temminck, 1858)	*T. tiptoni*
	S. asternalis
Sturnira erythromos (Tschudi 1844)	*A. phyllostomatis*
Sturnira hondurensis Goodwin, 1940	*A. delatorrei*
	M. proxima
	T. brennani
Sturnira lilium (E. Geoffroy, 1810)	*A. falcata*
	M. proxima
	T. phyllostomae
	Metelasmus wenzeli Graciolli & Dick, 2004

	T. joblingi<br>*T. uniformis*<br>*M. aranea*<br>*P. similis*<br>*A. phyllostomatis*<br>*S. guajiro*<br>*T. dugesioides dugesioides*<br>*P. longicrus*<br>*A. delatorrei*<br>*S. ambigua*<br>*Trichobius sp.*<br>*T. angulatus*<br>*T. spnov*
Sturnira ludovici Anthony, 1924	*T. longipes*<br>*M. proxima*
Sturnira parvidens Goldman, 1917	*M. proxima*<br>*A. delatorrei*
Sturnira tildae de la Torre, 1959	*A. falcata*<br>*M. proxima*<br>*T. parasiticus*<br>*M. aranea*<br>*P. dunni*
Tonatia bidens (Spix, 1823)	*S. galindoi*<br>*T. dugesioides dugesioides*<br>*S. mirabilis*<br>*T. dugesioides dugesioides*
Trachops cirrhosus (Spix, 1823)	*T. dugesioides dugesioides*<br>*T. joblingi*<br>*T. dugesioides*<br>*S. mirabilis*<br>*M. aranea*<br>*Speiseria magnioculus* Wenzel, 1976
Tonatia saurophila Koopman & Williams, 1951	*T. joblingi*<br>*S. galindoi*<br>*T. silvicolae*<br>*M. minuta*<br>*P. greenwelli*<br>*S. tonatiae*
Uroderma bilobatum Peters, 1866	*P. dunni*<br>*P. salvini*<br>*Paratrichobius sp.*
Vampyressa pusilla (Wagner, 1843)	*N. delicatus*<br>*T. dugesii*
