# Supplementary material for: Network and parasitological analyses reveal latitudinal gradient in bats‐ectoparasitic fly interactions across the Neotropic
Source: Ecol Evol. 2023 Sep 15;13(9):e10527. doi: 10.1002/ece3.10527 (PMC10502467; doi:10.1002/ece3.10527)
Supplement: Supplementary file 3 — Data S3 [file ECE3-13-e10527-s003.pdf]

Supplementary Data 3. Species of bats and their ectoparasitic flies and the respective codes used in the unified interaction network.

Bat Species	Code	Bat Flies Species	Code
A_caudifer	Ano_cau	T_tiptoni	Tri_tip
A_geoffroyi	Ano_geo	E_clovisi	Exa_clo
A_planirostris	Art_pla	S_harderi	Str_har
C_perpicillata	Car_per	M_aranea	Meg_ara
D_rotundus	Des_rot	T_joblingi	Tri_job
M_crenulatum	Gar_cre	S_guajiro	Str_gua
S_lilium	Stu_lil	S_wiedemanni	Str_wie
G_soricina	Glo_sor	B_tiptoni	Bas_tip
A_fimbriatus	Art_fim	P_longicrus	Par_lon
A_lituratus	Art_lit	A_falcata	Asp_fal
P_elongatus	Ply_elo	M_proxima	Meg_pro
T_cirrhosus	Tra_cir	T_dugesii	Tri_duge
L_aurita	Lon_aur	P_longipes	Par_long
U_bilobatum	Uro_bil	A_modestini	Ana_mod
S_tildae	Stu_til	A_caudiferae	Ana_cau
A_jamaicenses	Art_jam	M_pseudopterus	Met_pse
M_cf_nigricans	Myo_cf_nig	T_d_dugesioides	T_d_dug
P_lineatus	Pla_lin	T_d_phyllostomus	T_d_phy
P_hastatus	Phy_has	T_flagellatus	Tri_fla
A_obscurus	Art_obs	T_parasiticus	Tri_par
M_megalotis	Mic_meg	T_uniformis	Tri_uni
M_nigricans	Myo_nig	S_ambigua	Spe_amb
M_albescens	Myo_alb	P_dunni	Par_dun
M_ruber	Myo_rub	A_phyllostomatis	Asp_phy
M_levis	Myo_lev	S_consocia	Str_con
C_auritus	Chr_aur	S_altmani	Str_alt
E_brasiliensis	Ept_bra	B_ferrisi	Bas_fer
H_velatus	His_vel	T_lonchophyllae	Tri_lon
L_carrikeri	Lop_car	T_longipes	Tri_longi
M_minuta	Mic_min	T_phyllostomae	Tri_phy
P_discolor	Phy_dis	P_similis	Par_sim
L_brasiliense	Lop_bra	B_andersoni	Bas_and
T_bidens	Ton_bid	A_passosi	Ana_pas
L_peracchii	Lon_per	T_dugesioides	Tri_dug

D_cinerea	Der_cin	S_chrotopteri	Str_chro
V_pusilla	Vam_pus	M_wenzeli	Met_wen
P_recifinus	Pla_rec	B_plaumanni	Bas_pla
D_ecaudata	Dip_eca	B_ruiae	Bas_rui
L_silvicola	Lop_sil	M_minutaa	Mas_min
E_diminutus	Ept_dim	S_longirostris	Sti_lon
T_saurophila	Ton_sau	S_hertigi	Str_her
L_dekeyseri	Lon_dek	T_costalimai	Tri_cos
M_sanborni	Mic_san	T_perspicillatus	Tri_pers
P_stenops	Phy_ste	S_curvata	Str_cur
D_youngi	Dia_you	T_angulatus	Tri_ang
M_lavali	Myo_lav	B_quadrosae	Bas_qua
N_macrourus	Nat_mac	T_affinis	Tri_aff
F_horrens	Fur_hor	S_tonatiae	Str_ton
C_doriae	Chi_dor	S_mirabilis	Str_mir
L_mordax	Lon_mor	B_producta	Bas_pro
S_bilineata	Sac_bil	B_juquiensis	Bas_juq
N_albiventris	Noc_alb	S_hoogstraali	Str_hoo
M_riparius	Myo_rip	T_silvicolae	Tri_sil
E_furinalis	Ept_fur	P_sparsisetis	Pse_spa
P_gymnonotus	Pte_gym	P_greenwelli	Pse_gre
P_parnellii	Pte_par	P_riberoi	Pse_rib
C_brevicauda	Car_bre	B_mimoni	Bas_mim
H_thomasi	Hsu_tho	S_galindoi	Str_gal
L_spurrelli	Lio_spu	N_delicatus	Neo_del
M_bennettii	Mim_ben	S_diphyllae	Str_dip
R_pumilio	Rhi_pum	S_machadoi	Str_mac
T_nicefori	Tri_nic	T_anducei	Tri_and
R_naso	Rhy_nas	T_diphyllae	Tri_dip
N_leporinus	Noc_lep	T_furmani	Tri_fur
L_brachyotis	Lam_bra	T_handley	Tri_han
M_macrophyllum	Mac_mac	S_carvalhoi	Str_carv
S_erythromus	Stu_ery	T_propinquus	Tri_pro
G_daviesi	Gly_dav	B_ortizi	Bas_ort
C_castanea	Car_cas	A_delatorrei	Asp_del
D_bogotensis	Der_bog	T_bequaerti	Tri_beq
E_hartii	Enc_har	P_sanchezi	Par_san

P_helleri	Pla_hel	T_longipes	Tri_long
P_vittatus	Pla_vit	S_diaemi	Str_dia
S_ludovici	Stu_lud	T_diaemi	Tri_dia
M_oxyotus	Myo_oxy	B_travassosi	Bas_tra
A_latidens	Ano_lat	T_galei	Tri_gal
P_mesoamericanus	Pte_mes	T_pallidus	Tri_pal
M_cozumelae	Mim_coz	Trichobius_sp	Tri_sp
S_parvidens	Stu_par	P_parvula	Par_parv
N_mexicanus	Nat_mex	B_carteri	Bas_car
M_keaysi	Myo_kea	P_salvini	Par_sal
M_volans	Myo_vol	T_sp_parasiticus	T_sp_par
S_hondurensis	Stu_hon	T_sp_dugesii	T_sp_dug
C_sowelli	Car_sow	B_anceps	Bas_anc
P_davyi	Pte_dav	N_parnelli	Nyc_par
C_salvini	Chi_sal	T_persimilis	Tri_persi
L_yerbabuenae	Lep_yer	T_johnsonae	Tri_joh
M_megalophylla	Mor_meg	T_caecus	Tri_cae
P_personatus	Pte_per	S_peytonae	Spe_pey
G_morenoi	Glo_mor	T_lionycteridis	Tri_lio
M_microtis	Mic_mic	S_magnioculus	Spe_mag
L_evotis	Lop_evo	S_alvarezi	Str_alv
N_humeralis	Nyc_hum	S_obtusa	Str_obt
A_hirsutus	Art_hir	P_handleyi	Par_han
D_phaeotis	Der_pha	S_asternalis	Str_ast
D_tolteca	Der_tol	N_aitkeni	Noc_ait
C_subrufa	Car_sub	P_lineata	Par_lin
		N_bisetosus	Neo_bis
		S_christinae	Str_chr
		B_dunni	Bas_dun
		S_matsoni	Str_mat
		T_macrophylli	Tri_mac
		M_guimaraesi	M_gui
		P_parvuloides	Par_par
		N_maai	Noc_maa
		Basilia_sp.	Bas_sp
		T_perspicillatum	Tri_per
		Paratrichobius_sp	Par_sp

		E_oculatum	Exa_ocu
		N_coxata	Nyc_cox
		T_yunkeri	Tri_yun
		T_intermedius	Tri_int
		N_natali	Nyc_nat
		T_hirsutulus	Tri_hir
		A_scorzai	Ana_sco
		T_brennani	Tri_bre
		S_carollinae	Str_car
		P_lowei	Par_low
		T_sparsus	Tri_spa
		T_sphaeronotus	Tri_sph
		N_traubi	Noc_tra
		T_hoffmannae	Tri_hof
		S_kohlsi	Str_koh
		N_fairchildi	Nyc_fair
		T_leionotus	Tri_lei
		B_hughscotti	Bas_hug
		T_spnov	T_spnov
		B_lindolphoi	Bas_lin
